# Editorial: Advanced therapeutic strategies in digestive and circulatory health: nanomedicine and beyond

**DOI:** 10.3389/fphar.2025.1666774

**Published:** 2025-07-31

**Authors:** Dan Li, Peng Zhang, Hui Chen

**Affiliations:** ^1^ School of Pharmacy, Jinzhou Medical University, Jinzhou, China; ^2^ Biomedical Engineering Lab, Politecnico di Torino, Turin, Italy; ^3^ Department of Applied Science and Technology, Politecnico di Torino, Turin, Italy; ^4^ Laboratory of Chemical Oncogenomics, Institute of Biomedical and Health Engineering, Shenzhen International Graduate School, Tsinghua University, Shenzhen, China; ^5^ Guangzhou Key Laboratory of Analytical Chemistry for Biomedicine, School of Chemistry, South China Normal University, Guangzhou, China

**Keywords:** advanced therapeutic strategies, digestive, circulatory, nanomedicine, beyond

Diseases of the digestive system and related circulatory networks significantly impact human health, leading to high rates of illness and death worldwide. Conditions such as colorectal cancer, liver dysfunction, and cardiovascular complications not only reduce quality of life but also create substantial social and economic burdens. Innovative approaches—including nanomedicine, new pharmacological agents, and integrated diagnostics—are enabling earlier detection, targeted treatments, and improved outcomes. This editorial highlight five recent studies in Frontiers in Pharmacology, showcasing advances from colorectal cancer diagnostics to novel drug delivery systems and risk assessments, all reflecting the ongoing shift toward precision and personalized medicine in digestive and circulatory health.

**FIGURE 1:**
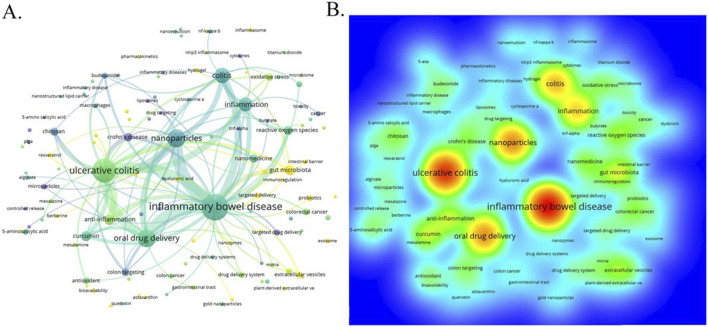
**(A)** The visualization map of keywords co-occurrence network on nanomedicine applications in IBD. **(B)** The heatmap of keywords.

Nanomedicine, as a transformative paradigm, is increasingly central to therapeutic innovations in digestive health, particularly for conditions like inflammatory bowel disease (IBD). The comprehensive bibliometric analysis by Jiang et al. map the intellectual structure and emerging trends in nanomedicine applications for IBD, identifying key research hotspots such as targeted drug delivery, immune cell regulation, and intestinal microbiota homeostasis. Their study, spanning over 2 decades of global research, reveals the rapid growth and interdisciplinary nature of this field, with China emerging as a leading contributor (Jiang et al.). To visually encapsulate the depth of this analysis, we propose the inclusion of a figure (Figure 1) at this juncture. This visual representation underscores the interconnectedness of research themes and the evolving focus on precision and safety in nanomedicine, directly contributing to the overarching Research Topic by providing a roadmap for future investigations and clinical translations in digestive health.

One of the most formidable challenges in digestive health lies in the accurate diagnosis and classification of colorectal lesions, a critical step in combating colorectal cancer, which ranks as the third most prevalent malignancy globally. The work by Fu *et al* introduces a groundbreaking multimodal framework termed Pathology-Attention Multi-Instance Learning (PAT-MIL), which integrates dynamic attention mechanisms with pathology-driven text prototypes to achieve superior classification of whole slide images (WSIs). This innovative approach not only addresses the limitations of weakly supervised learning by modeling the collaborative diagnostic process of pathologists but also mitigates Research Topic such as staining variability and tissue heterogeneity. With reported accuracies of up to 86.45% on internal datasets and significant improvements over existing baselines, PAT-MIL exemplifies how multimodal strategies can enhance diagnostic precision and generalization across diverse clinical settings (Fu et al.). This contribution is pivotal to the Research Topic of advanced therapeutic strategies, as it lays the foundation for more reliable early detection and tailored interventions in digestive malignancies.

In the realm of cancer therapy, which intersects both digestive and systemic health, the development of novel drug delivery systems remains a cornerstone of therapeutic advancement. Wang et al. present a compelling study on the use of Poly (trimethylene carbonate) (PTMC) membranes for the sustained release of 5-Fluorouracil (5-FU), a widely used chemotherapeutic agent. Their findings reveal that these membranes not only ensure prolonged drug release but also exhibit superior biocompatibility and enhanced antitumor efficacy compared to conventional injections. This innovation holds immense potential for improving chemotherapy outcomes in cancer patients by minimizing systemic toxicity and optimizing therapeutic impact (Wang et al.). Within the broader theme of advanced therapeutic strategies, this work exemplifies how material science can revolutionize drug delivery.

In the realm of liver transplantation, Liu et al. present compelling evidence for the efficacy of Rutaecarpine (Rut) in mitigating hepatic ischemia-reperfusion injury (IRI), a major complication in donation after circulatory death (DCD) liver grafts. Their research demonstrates that Rut not only reduces liver dysfunction and tissue damage but also significantly improves survival in rat transplantation models. Mechanistically, Rut’s protective effects are linked to its inhibition of PDE4B, a pivotal regulator of inflammation and oxidative stress. These findings highlight Rut’s potential as an innovative therapeutic agent to address IRI and improve outcomes for liver transplant recipients (Liu et al.).This research enriches the discourse on advanced therapeutic strategies by highlighting the intersection of pharmacological innovation and circulatory health, offering a pathway to mitigate one of the critical barriers to successful transplantation outcomes.

Lastly, the intersection of therapeutic safety and efficacy is critically examined in the study by Qiao et al., who analyze the risk of oncological adverse events associated with infliximab and azathioprine, both as monotherapies and in combination, using data from the FDA Adverse Event Reporting System (FAERS). Their findings suggest that combination therapy does not significantly elevate tumor development risk compared to azathioprine alone, and may even reduce such risks in patients on infliximab monotherapy. This nuanced analysis provides essential insights into the safety profiles of commonly used drugs in digestive health conditions like IBD, ensuring that therapeutic strategies remain balanced between efficacy and risk mitigation (Qiao et al.). This work is a vital component of advanced therapeutic strategies, as it informs clinical decision-making and underscores the importance of pharmacovigilance in long-term treatment plans.

Collectively, these five studies illuminate the multifaceted nature of advanced therapeutic strategies in digestive and circulatory health, spanning nanomedicine trends, diagnostics, drug delivery innovations, pharmacological interventions, and safety assessments. Each contribution adds a unique dimension to the overarching theme of “Advanced Therapeutic Strategies in Digestive and Circulatory Health: Nanomedicine and Beyond.” However, this theme still faces challenges such as limited clinical translation, long-term safety uncertainties, and insufficient consideration of patient diversity. Future research may focus on large-scale validation, standardized safety evaluation, and improving accessibility, ensuring that technological innovation truly benefits diverse patient populations.

As we stand at the cusp of a new era in medical science, these studies collectively challenge us to rethink traditional paradigms and embrace innovation. They call for continued investment in research that bridges the gap between bench and bedside, ensuring that the promise of advanced therapeutic strategies translates into tangible benefits for patients worldwide. We invite researchers, clinicians, and policymakers to engage with these findings, fostering collaborations that will drive the next wave of breakthroughs in this vital field.

